# Leaf-age and petiole biomass play significant roles in leaf scaling theory

**DOI:** 10.3389/fpls.2023.1322245

**Published:** 2023-12-21

**Authors:** Xuchen Guo, Julian Schrader, Peijian Shi, Yabing Jiao, Qinyue Miao, Jianhui Xue, Karl J. Niklas

**Affiliations:** ^1^ Co-Innovation Centre for Sustainable Forestry in Southern China, Bamboo Research Institute, College of Biology and Environment, Nanjing Forestry University, Nanjing, China; ^2^ School of Natural Sciences, Macquarie University, Sydney, NSW, Australia; ^3^ Institute of Botany, Jiangsu Province and Chinese Academy Sciences, Nanjing, China; ^4^ School of Integrative Plant Science, Cornell University, Ithaca, NY, United States

**Keywords:** diminishing returns, leaf mass per unit area, ontogeny, scaling growth, trade-offs

## Abstract

Foliage leaves are essential for plant survival and growth, and how plants allocate biomass to their leaves reveals their economic and ecological strategies. Prior studies have shown that leaf-age significantly influences leaf biomass allocation patterns. However, unravelling the effects of ontogeny on partitioning biomass remains a challenge because it is confounded by the effects of environmental factors. Here, we aim to elucidate whether leaf-age affects the allocation to the lamina and petiole by examining leaves of known age growing in the same general environmental context. We sampled 2698 *Photinia serratifolia* leaves developing in the same environment from April to November 2021, representing eight leaf-ages (*n* > 300 for each leaf-age). Petiole and lamina biomass, and lamina area were measured to evaluate the scaling relationships using reduced major axis regression protocols. The bootstrap percentile method was used to determine the differences in scaling exponents among the different leaf-ages. ANOVA with Tukey’s HSD was used to compare the ratios of petiole and lamina biomass to lamina area across the leaf-ages. Correlation tests were used to determine if exponents, intercepts, and ratios differed significantly across the different leaf-ages. The data indicated that (i) the ratio of petiole and lamina biomass to lamina area and the scaling exponent of lamina biomass versus lamina area correlate positively with leaf-age, and (ii) the scaling exponent of petiole biomass versus lamina area correlates negatively with leaf-age. Leaf maturation process involves an inverse proportional allocation between lamina and petiole biomass for expanding photosynthetic area. This phenomenon underscores the effect of leaf-age on biomass allocation and the importance of adopting an ontogenetic perspective when entertaining plant scaling theories and unravelling the principles governing shifts in biomass allocation throughout the leaf lifespan.

## Introduction

Foliage leaves are the primary photosynthetic organs of terrestrial plants and therefore are critical to plant survival and growth (Rascher and Nedbal, 2006; Díaz et al., 2016; Adams III and Terashima, 2018). The photosynthates produced by leaves are utilized to sustain metabolism, support the functions of other organs, and regenerate damaged body parts (Nakamura and Hashimoto, 1988; Lockhart et al., 2003; Wright et al., 2004). However, the availability of raw materials for photosynthesis constrains the production of photosynthates, prompting plants to balance the allocation of resources to leaves, stems, and roots according to the physiological demands of each organ-type (Enquist and Niklas, 2002; Cheng et al., 2015). Prior physiological studies using different measurement methods provide insights into the utilization and transportation of photosynthates (Farrar and Jones, 2000; Minchin and Thorpe, 2003; Jahnke et al., 2009), and the final metabolic outcome of these processes can be assessed by measuring the biomass allocated to each organ- or tissue-type (Poorter et al., 2012).

Eudicot leaves are composed of two basic structural units, the lamina and the petiole, and the distribution of biomass between these two different units reflects a trade-off between their respective functions (Niklas, 1992; Niklas, 1999; Niklas and Enquist, 2002; Pasini and Mirjalili, 2006). This distribution, referred to as “leaf biomass allocation”, provides valuable information about plant economics and ecological strategies, and its variations reveal the ecological and evolutionary processes that shape plant development (Niklas, 1994; Westoby et al., 2002; Niklas et al., 2007).

Previous studies have applied two approaches (i.e., the ratio and scaling approach) to characterize leaf biomass allocation (Poorter et al., 2012; Poorter et al., 2015). The first approach involves determining the quotient of biomass at a specific time to describe the allocation of biomass to a particular structural or functional trait, such as lamina dry mass per unit area (LMA), which represents the leaf-level cost for light-harvesting (Poorter et al., 2009). Wright et al. (2005) quantified climatic influences on key leaf traits globally and reported that plants tend to have higher mean LMA at sites experiencing greater environmental stress (e.g., hotter, drier, and higher irradiance sites).

In contrast, the scaling approach employs a power-law equation to describe the relationship between or among different body parts (Niklas, 1994; Enquist et al., 2007; Niinemets et al., 2007; Niklas et al., 2009; Sack et al., 2012). For example, the lamina biomass (*M*) and area (*A*) scaling relationship exhibits a log-log linear relationship with a scaling exponent (i.e., the log-log slope denoted by α) whose numerical value reflects different leaf biomass allocation patterns affecting light interception and mechanical support. An isometric scaling relationship (i.e., α = 1.0) indicates that increases in leaf biomass obtain a one-to-one proportional increase in area (i.e., M∝A ). However, Niklas et al. (2007) found that the numerical value of the interspecific scaling exponent often exceeds unity (i.e., α > 1.0), indicating that increases in lamina biomass do not obtain proportional increases in lamina area. This phenomenon has been called “diminishing returns” (also see Milla and Reich, 2007).

However, a limited number of studies have tested leaf-age’s effects on biomass allocation patterns. Jiao et al. (2022) reported that the numerical value of the scaling exponent α increases as *Photinia* × *fraseri* “Red Robin” leaves grow older, resulting in a higher *M* vs. *A* scaling exponent for leaves sampled in the summer compared to leaves sampled in the spring. However, the proximate cause for the phenomenon was not resolved because it can be explained by seasonal changes in environmental factors (i.e., temperature and precipitation significantly increase in the summer) as well as developmental changes in leaf functional traits (Reich et al., 2014) and leaf growth and structural allocation patterns (Hudson et al., 2011). Therefore, the effect of leaf-age on leaf biomass allocation patterns remains uncertain due to a paucity of direct observation and phenotypic plasticity in response to environmental factors (Jiao et al., 2022; Westoby et al., 2022).

To address this area of uncertainty, we collected the leaves of the evergreen species *Photinia serratifolia* (Desfontaines) Kalkman in eight leaf-age groups from the spring to the winter of 2021. P*. serratifolia* was selected due to the new leaves of this species only emerged on the top of branches once a year, there is no need to worry about the investigated leaves being shadowed by another round of new leaves. We employed both the ratio and scaling approaches to investigate the relationships between lamina biomass and area. In addition, the scaling relationships between petiole biomass and other variables of interest (e.g., lamina biomass and lamina area) of the eight different age groups were examined because of their important mechanical and hydraulic functionalities (Sack et al., 2003; Filartiga et al., 2022; Li et al., 2022). The goal of this study was to answer one key question: does leaf-age affect leaf biomass allocation patterns?

## Materials and methods

### Leaf collection

**Figure 1:**
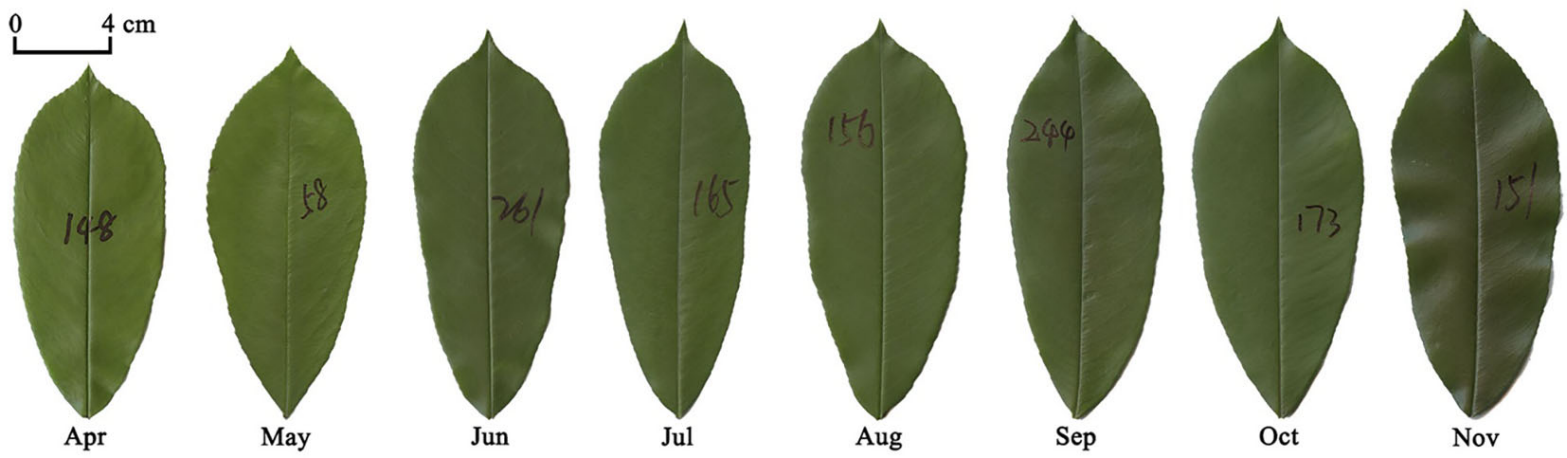
Examples of the lamina of eight leaf-age groups investigated in this study.

Newly emerging leaves on three *P. serratifolia* trees, which all grew in Nanjing Forestry University campus (118°48′35″E, 32°4′67″N), Nanjing, Jiangsu Province, China, were tagged during late February and early March in 2021, and a total of 2698 leaves were collected from April to November, with over 300 leaves sampled for each month. **Table 1** provides the sampling information for each leaf-age group, and **Figure 1** presents representative examples of the investigated leaf-age groups. **Supplementary Figure S1** presents examples of the investigated tagged leaves. The climatic information pertinent to **Table 1** was collected from https://www.ncei.noaa.gov (Station Name: NANJING; ID: CHM00058238; 118.9°E, 31.93°N; 15 m a.s.l.).

**Table 1:** Sampling months and relevant climatic information.

Leaf-age (months)	Monthly mean temperature (°C)	Monthly precipitation (mm)	Sampling time
1	16.26	46.99	Apr 16th
2	22.15	225.04	May 14th
3	26.07	77.72	Jun 17th
4	28.19	385.32	Jul 15th
5	27.49	199.90	Aug 16th
6	26.18	38.86	Sep 17th
7	18.92	120.39	Oct 18th
8	12.11	23.11	Nov 19th

### Leaf measurements

Leaves were scanned to bitmap images at a 600-dpi resolution using a photo scanner (V550, Epson Indonesia, Batam, Indonesia). Adobe Photoshop CS6 (version: 13.0; Adobe, San Jose, CA, USA) was used to obtain lamina black-white images. The planar coordinates of each lamina boundary were extracted using an M-file based on MATLAB (version ≥ 2009a; MathWorks, Natick, MA, USA) developed by Shi et al. (2018). The lamina area of each leaf was calculated using the “bilat” function in the “biogeom” package (version 1.3.5; Shi et al., 2022) using R software (version 4.2.0; R Core Team, 2022). Lamina fresh mass (LFM) and dry mass (LDM), and petiole fresh mass (PFM) and dry mass (PDM) were measured using an electronic balance (Type: ML 204; Mettler Toledo Company, Greifensee, Switzerland). LFM and PFM were determined after drying laminae and petioles to constant weight in an oven (Type: XMTD8222; Jinghong Experimental Equipment Co., Ltd., Shanghai, China) at 80°C for 48 h before measurement.

### Statistical methods

Preliminary regression analyses of the untransformed and log-transformed data indicated that scaling relationships statistically complied with power-law functions taking the general form of:


(1) $$Y_{1}=\text{β}Y_{2}^{\text{α}},$$

where $Y_{1}$ and $Y_{2}$ represent two interdependent variables of interest (e.g., lamina dry mass and petiole dry mass), and α and β are the slope and *y*-intercept of the log-log regression curve (i.e., the scaling exponent and the normalization constant, respectively), respectively. The log-transformed form of Equation 1 takes the linear form (Niklas, 1994; Niklas et al., 2007).


(2) y=γ+αx,

where $y=\text{ln}\left(Y_{1}\right)$ , $x=\text{ln}\left(Y_{2}\right)$ , and γ=ln(β) . Parameters γ and α in Equation 2 were estimated using reduced major axis regression protocols (Niklas, 1994; Smith, 2009). The bootstrap percentile method (using 3000 bootstrapping replicates) was used to test the significance of the difference in the estimated scaling exponents of y vs. x between any two of the leaf-age groups (Efron and Tibshirani, 1993; Sandhu et al., 2011). Analysis of variance followed by Turkey’s honestly significant difference test with a 0.05 significance level were used to test for the significance of the differences in the quotients of LDM and *A*, PDM and *A*, LFM and *A*, and PFM and *A* among eight leaf-age groups (Hsu, 1996). Correlation tests were used to test whether there were significant correlations between the slopes (i.e., the scaling exponents) and leaf-age, between the intercepts and leaf-age, and between the ratios of leaf traits and leaf-age. All statistical analyses were performed using R (version 4.2.0; R Core Team, 2022).

## Results

The numerical values of the scaling exponents of LDM vs. *A* were significantly greater than unity (i.e., α > 1.0) for each of the seven leaf-age groups, i.e., the lower bounds of the corresponding 95% confidence intervals (CIs) exceeded unity, with the exception of the first age group whose 95% CIs did include unity. Similarly, the scaling exponents of PDM vs. *A* were significantly greater than unity for the first and second age groups, whereas the 95% CIs of the scaling exponents for the remaining six age-groups included unity or the upper bounds of the 95% CIs were significant smaller than unity. The goodness of fit for LDM vs. *A* was significantly better than that of PDM vs. *A*. The *r* ^2^ values for LDM vs. *A* were typically greater than 0.8, with two exceptions (i.e., 0.735 and 0.793), whereas the *r* ^2^ for PDM vs. *A* was consistently smaller than 0.8 (**Table 2**).

**Table 2:** Fitted results for lamina dry mass (LDM) vs. lamina area (*A*) and petiole dry mass (PDM) vs. *A* in eight leaf-age groups.

Leaf-age (months)	Sample size	Scaling relationship	Fitted equation	95% confidence interval of the slope	95% confidence interval of the intercept	*r* ^2^
1	332	LDM vs. *A*	*y* = –4.707 + 1.026 *x*	(0.979, 1.078)	(-4.917, -4.514)	0.817
		PDM vs. *A*	*y* = –7.663 + 1.098 *x*	(1.033, 1.169)	(-7.958, -7.397)	0.568
2	335	LDM vs. *A*	*y* = –4.678 + 1.047 *x*	(1.004, 1.091)	(-4.865, -4.498)	0.828
		PDM vs. *A*	*y* = –7.342 + 1.049 *x*	(1.004, 1.092)	(-7.524, -7.156)	0.789
3	347	LDM vs. *A*	*y* = –4.783 + 1.090 *x*	(1.046, 1.136)	(-4.974, -4.600)	0.835
		PDM vs. *A*	*y* = –7.091 + 1.030 *x*	(0.961, 1.104)	(-7.403, -6.799)	0.587
4	331	LDM vs. *A*	*y* = –4.498 + 1.045 *x*	(1.004, 1.089)	(-4.682, -4.328)	0.858
		PDM vs. *A*	*y* = –6.585 + 0.934 *x*	(0.883, 0.990)	(-6.816, -6.376)	0.705
5	325	LDM vs. *A*	*y* = –4.633 + 1.075 *x*	(1.026, 1.125)	(-4.836, -4.439)	0.842
		PDM vs. *A*	*y* = –6.860 + 1.008 *x*	(0.955, 1.066)	(-7.093, -6.646)	0.680
6	345	LDM vs. *A*	*y* = –4.808 + 1.111 *x*	(1.053, 1.170)	(-5.049, -4.565)	0.735
		PDM vs. *A*	*y* = –6.905 + 0.968 *x*	(0.932, 1.041)	(-7.136, -6.674)	0.753
7	346	LDM vs. *A*	*y* = –4.904 + 1.136 *x*	(1.092, 1.181)	(-5.095, -4.718)	0.793
		PDM vs. *A*	*y* = –6.588 + 0.937 *x*	(0.885, 0.997)	(-6.843, -6.364)	0.586
8	337	LDM vs. *A*	*y* = –4.952 + 1.143 *x*	(1.092, 1.198)	(-5.176, -4.735)	0.810
		PDM vs. *A*	*y* = –6.869 + 0.999 *x*	(0.932, 1.068)	(-7.165, -6.587)	0.498
Pool data	2698	LDM vs. *A*	*y =* − 4.901 + 1.123*x*	(1.102, 1.145)	(-4.993, -4.815)	0.760
		PDM vs. *A*	*y =* − 7.433 + 1.114*x*	(1.086, 1.144)	(-7.558, -7.315)	0.492

The scaling exponents of LFM vs. *A* were significantly greater than unity for all leaf-age groups, with the lower bounds of the corresponding 95% CIs exceeding unity in each age group. The 95% CIs of the scaling exponents of PFM vs. *A* included unity for the third, seventh, and eighth leaf-age groups; for the remaining age groups, the scaling exponents of PFM vs. *A* were significantly greater than unity, i.e., the lower bounds of the 95% CIs were greater than unity. The goodness of fit for LFM vs. *A* was significantly greater than that of PFM vs. *A*. The *r* ^2^ values for LFM vs. *A* were consistently above 0.9, whereas the *r* ^2^ values for PFM vs. *A* did not exceed 0.8 (**Table 3**). The data indicated that the scaling exponents of LDM vs. *A* tended to be slightly greater than those of LFM vs. *A*, and that the exponents of PDM vs. *A* tended to be slightly smaller than those of PFM vs. *A*. Additionally, the goodness of fit for both lamina fresh mass and petiole fresh mass was better than for their corresponding dry mass counterparts (**Tables 2**, **3**).

**Table 3:** Fitted results for lamina fresh mass (LFM) vs. lamina area (*A*) and petiole fresh mass (PFM) vs. *A* in eight leaf-age groups.

Leaf-age (months)	Sample size	Scaling relationship	Fitted equation	95% confidence interval of the slope	95% confidence interval of the intercept	*r* ^2^
1	332	LFM vs. *A*	*y* = –3.707 + 1.031 *x*	(1.002, 1.064)	(-3.840, -3.587)	0.948
		PFM vs. *A*	*y* = –6.962 + 1.205 *x*	(1.137, 1.279)	(-7.264, -6.684)	0.565
2	335	LFM vs. *A*	*y* = –3.748 + 1.043 *x*	(1.020, 1.066)	(-3.845, -3.652)	0.958
		PFM vs. *A*	*y* = –6.946 + 1.208 *x*	(1.159, 1.260)	(-7.164, -6.742)	0.743
3	347	LFM vs. *A*	*y* = –3.831 + 1.069 *x*	(1.041, 1.099)	(-3.957, -3.716)	0.933
		PFM vs. *A*	*y* = –6.223 + 1.062 *x*	(0.997, 1.129)	(-6.506, -5.955)	0.638
4	331	LFM vs. *A*	*y* = –3.640 + 1.032 *x*	(1.007, 1.057)	(-3.745, -3.540)	0.954
		PFM vs. *A*	*y* = –6.215 + 1.061 *x*	(1.001, 1.126)	(-6.486, -5.962)	0.639
5	325	LFM vs. *A*	*y* = –3.687 + 1.045 *x*	(1.013, 1.079)	(-3.826, -3.556)	0.948
		PFM vs. *A*	*y* = –6.376 + 1.130 *x*	(1.064, 1.205)	(-6.679, -6.107)	0.579
6	345	LFM vs. *A*	*y* = –3.772 + 1.057 *x*	(1.024, 1.089)	(-3.905, -3.635)	0.922
		PFM vs. *A*	*y* = –6.306 + 1.066 *x*	(1.023, 1.108)	(-6.486, -6.125)	0.786
7	346	LFM vs. *A*	*y* = –3.883 + 1.081 *x*	(1.055, 1.109)	(-4.004, -3.772)	0.924
		PFM vs. *A*	*y* = –5.886 + 0.995 *x*	(0.940, 1.057)	(-6.151, -5.655)	0.582
8	337	LFM vs. *A*	*y* = –3.987 + 1.122 *x*	(1.074, 1.151)	(-4.158, -3.832)	0.910
		PFM vs. *A*	*y* = –5.962 + 1.020 *x*	(0.958, 1.085)	(-6.238, -5.702)	0.597
Pool data	2698	LFM vs. *A*	*y =* − 3.761 + 1.054 *x*	(1.042, 1.066)	(-3.810, -3.714)	0.936
		PFM vs. *A*	*y =* − 6.550 + 1.139 *x*	(1.116, 1.164)	(-6.655, -6.453)	0.597

**Figure 2:**
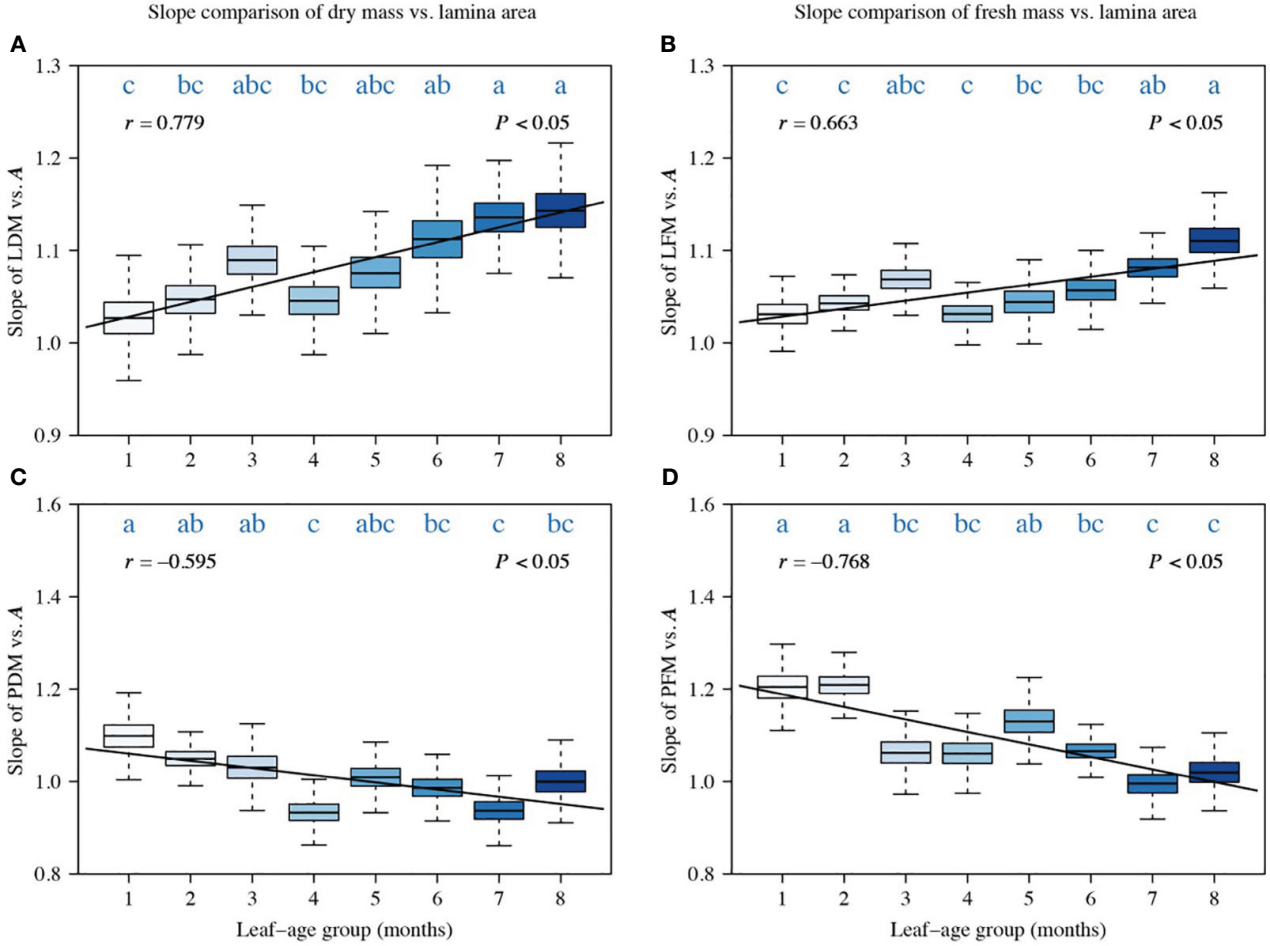
Comparisons of the scaling exponents (α-values) of LDM vs. *A* **(A)**, of LFM vs. *A* **(B)**, of PDM vs. *A* **(C)**, and of PFM vs. *A* **(D)**. Each boxplot was obtained from 3000 bootstrap replications. The lowercase letters a–c on the top of each box denote the significance of the difference in the scaling exponents between any two leaf-ages at a 0.05 significance level. *r* is the correlation coefficient for the scaling exponents and leaf-age groups, and *P* is the significance test parameter. Leaf-age codes correspond to those in **Table 1**.

A comparison of the scaling exponents of LDM vs. *A*, PDM vs. *A*, LFM vs. *A*, and PFM vs. *A* indicated a significant correlation between the scaling exponents and leaf-age (|*r*| > 0.5, *P* < 0.05). Additionally, the temporal variation in the lamina mass vs. *A* scaling exponent and that in the petiole mass vs. *A* scaling exponent were significantly opposite (**Figure 2**). The scaling exponents of LDM vs. *A*, and LFM vs. *A* numerically increased with increasing leaf-age, and the positive correlation between the LDM vs. *A* scaling exponent and leaf-age was more robust than that of the LFM vs. *A* scaling exponent, as indicated by a larger correlation coefficient (**Figures 2A, B**). Conversely, the scaling exponents of PDM vs. *A*, and PFM vs. *A* decreased with increasing leaf-age, and the negative correlation between PFM vs. *A* scaling exponent and leaf-age was more robust than that between the PDM vs. *A* scaling exponent and leaf-age, as reflected by a lower correlation coefficient (**Figures 2C, D**). Although there were slight differences in the scaling exponents of LDM vs. *A* and LFM vs. *A*, the influence of leaf-age on those exponents was similar. The same held true for petiole scaling exponents that vary in the opposite direction (**Figure 2**).

**Figure 3:**
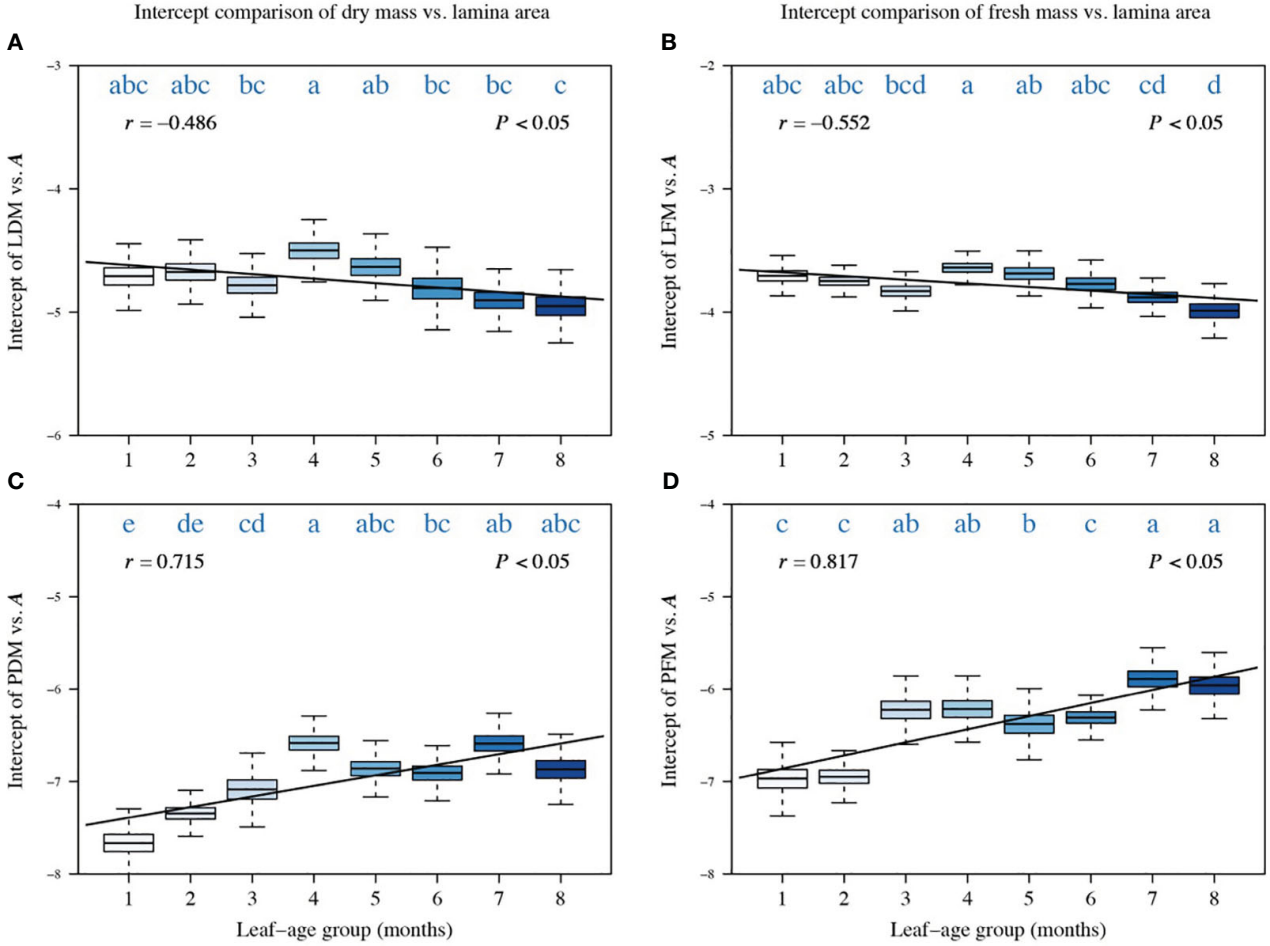
Comparisons of the intercepts of LDM vs. *A* **(A)**, of LFM vs. *A* **(B)**, of PDM vs. *A* **(C)**, and of PFM vs. *A* **(D)**. Each boxplot was obtained from 3000 bootstrap replications. The lowercase letters a–c on the top of each box denote the significance of the difference in the scaling exponents between any two leaf-ages at a 0.05 significance level. *r* is the correlation coefficient for the scaling exponents and leaf-age groups, and *P* is the significance test parameter. Leaf-age codes correspond to those in **Table 1**.

**Figure 4:**
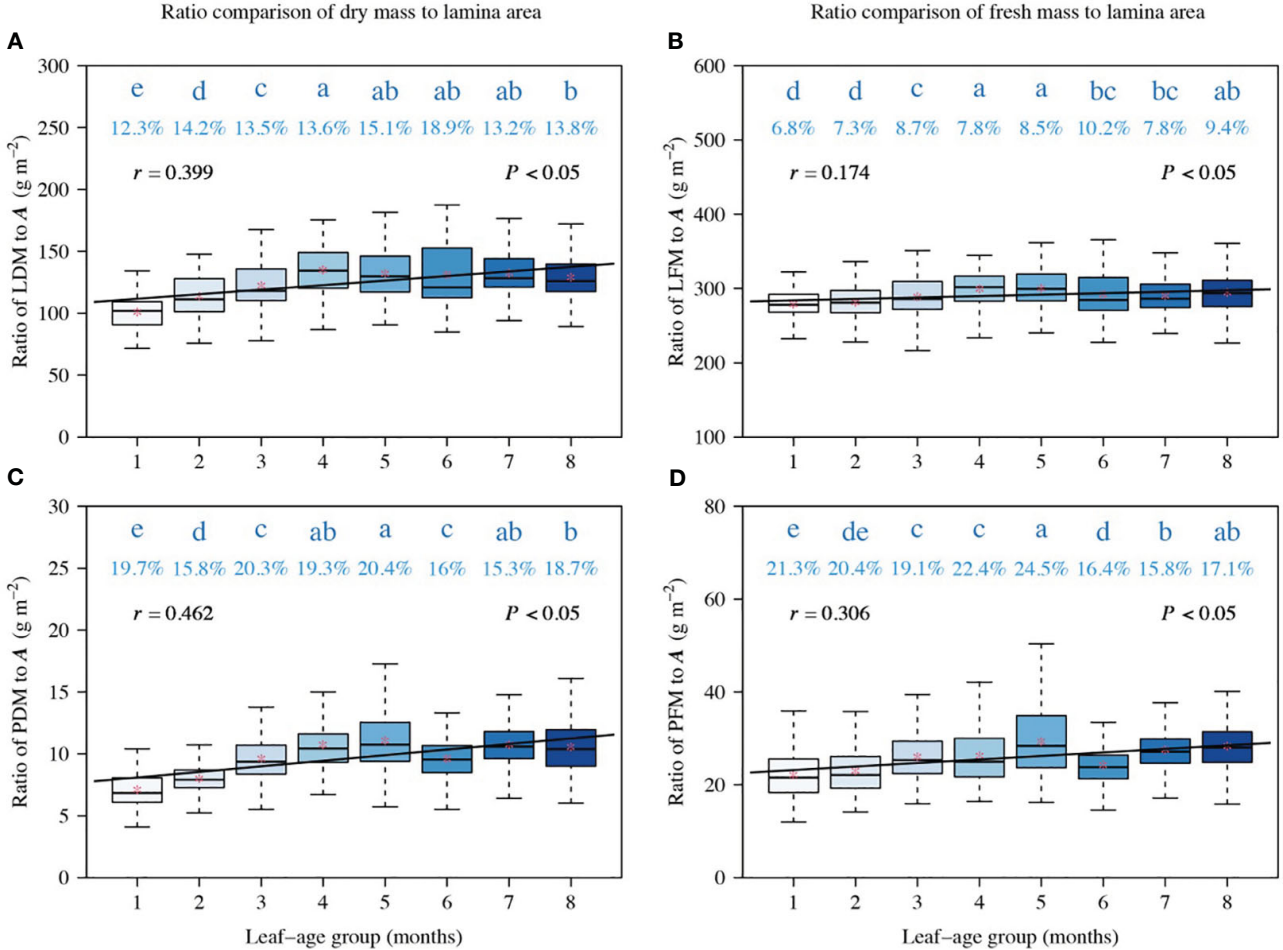
Comparisons of the ratios of LDM to *A* **(A)**, LFM to *A* **(B)**, PDM to *A* **(C)**, and PFM to *A* **(D)**. The lowercase letters a–e on the top of each box denote the significance of the difference in the means between any two leaf-ages based on Tukey’s HSD test at a 0.05 significance level. The numbers above the whiskers represent the coefficients of variation (%). The horizontal solid lines represent the medians, and the asterisks within boxes represent the means. *r* is the correlation coefficient for the scaling exponents and leaf-age groups, and *P* is the significance test parameter. Leaf-age codes correspond to those in **Table 1**.

A comparison of the intercepts of PDM vs. *A*, LFM vs. *A*, and PFM vs. *A* also reveals a significant correlation between the intercept and leaf-age (|*r*| > 0.5, *P* < 0.05), with the exception of the intercept of LDM vs. *A* whose correlation coefficient with leaf-age is −0.481 (**Figure 3**). The trend in the variation of the intercept was opposite to the trend in the numerical value of the scaling exponent (**Figures 3**, **4**). This inverse relationship has been reported previously for other scaling relationship but is not always biologically meaningful because it emerges as a mathematical artifact whenever multiple scaling relationships share a common point ( $Y_{1}$ , $Y_{2}$ ) for which $Y_{2}>1\;\;$ (Niklas and Hammond, 2019). Nevertheless, the observed variation in the numerical values of intercepts is meaningful because the values influence the quantities of biomass allocated to the construction of laminas given that $M=\text{β}A^{\text{α}}$ . In addition, the correlations between the intercept of LDM vs. *A* and leaf-age, and between PDM vs. *A* and leaf-age (**Figures 3A, C**) were less robust than their fresh mass counterparts (**Figures 3B, D**), as shown by the numerically larger or smaller correlation coefficients.

The ratios of LDM to *A*, LFM to *A*, PDM to *A*, and PFM to *A* were correlated positively with leaf-age (*P* < 0.05), and the correlation between the ratios of LFM to *A* and leaf-age was less statistically robust than the other correlations (**Figure 4**). Additionally, the positive correlation between ratios of dry mass (including lamina dry mass and petiole dry mass) to lamina area (**Figures 4A, C**) and leaf-age were more robust than their fresh mass counterparts (**Figures 4B, D**), as shown by the numerically larger correlation coefficients.

## Discussion

This study has documented that there is a difference between the ratio and scaling approaches. As determined by the ratio approach, both the ratio of lamina biomass to lamina area and the ratio of petiole biomass to lamina area increased with increasing leaf-age. In contrast, the scaling approach reveals a statistically significant opposite trend in the scaling exponents dictating lamina biomass vs. lamina area compared to that of petiole biomass vs. lamina area. In this context, it is worth noting that the same ratio can be achieved by either a decrease in the denominator or an increase in the numerator, which makes the interpretation of a ratio potentially ambiguous when taken in isolation. Consequently, it is wise to consider the results of the ratio and scaling approach in tandem to eliminate any potential ambiguities in interpreting results such as those presented here.

Based on these two approaches, this study confirms that leaf-age is an important factor in determining the scaling relationships of leaf biomass allocation patterns. However, previous studies have attributed the variation of leaf biomass allocation patterns to environmental factors. For example, Pan et al. (2013) showed that the numerical value of α increases with altitude across 121 vascular plant species. Likewise, Thakur et al. (2019) observed that the numerical value of α increases with the degree of environmental stress (i.e., higher, drier, open habitats). These studies indicate that leaf development is responsive to local ambient conditions in addition to differing across species. In contrast, our data indicate that environmental differences during the eight months over which leaf were sampled had little or no observable effect. The correlation test of the relationships between the numerical values of the scaling exponents and normalization constants for any of the biomass allocation patterns for lamina and petiole (listed in **Tables 2**, **3**) and the differences in monthly temperature and precipitation among the eight leaf samples (listed on **Table 1**) failed to reveal any statistically significant relationship (i.e., *P >*0.05). Although it is important to note in this context that the absence of evidence for an “environmental effect” is based on a small sample size of leaf-age (*n* = 8) drawn from only one taxon (see **Supplementary Figures S2** and **S3**). In addition, the differences in the monthly temperature or precipitation recorded over the duration of sampling are arguably not extreme.

**Figure 5:**
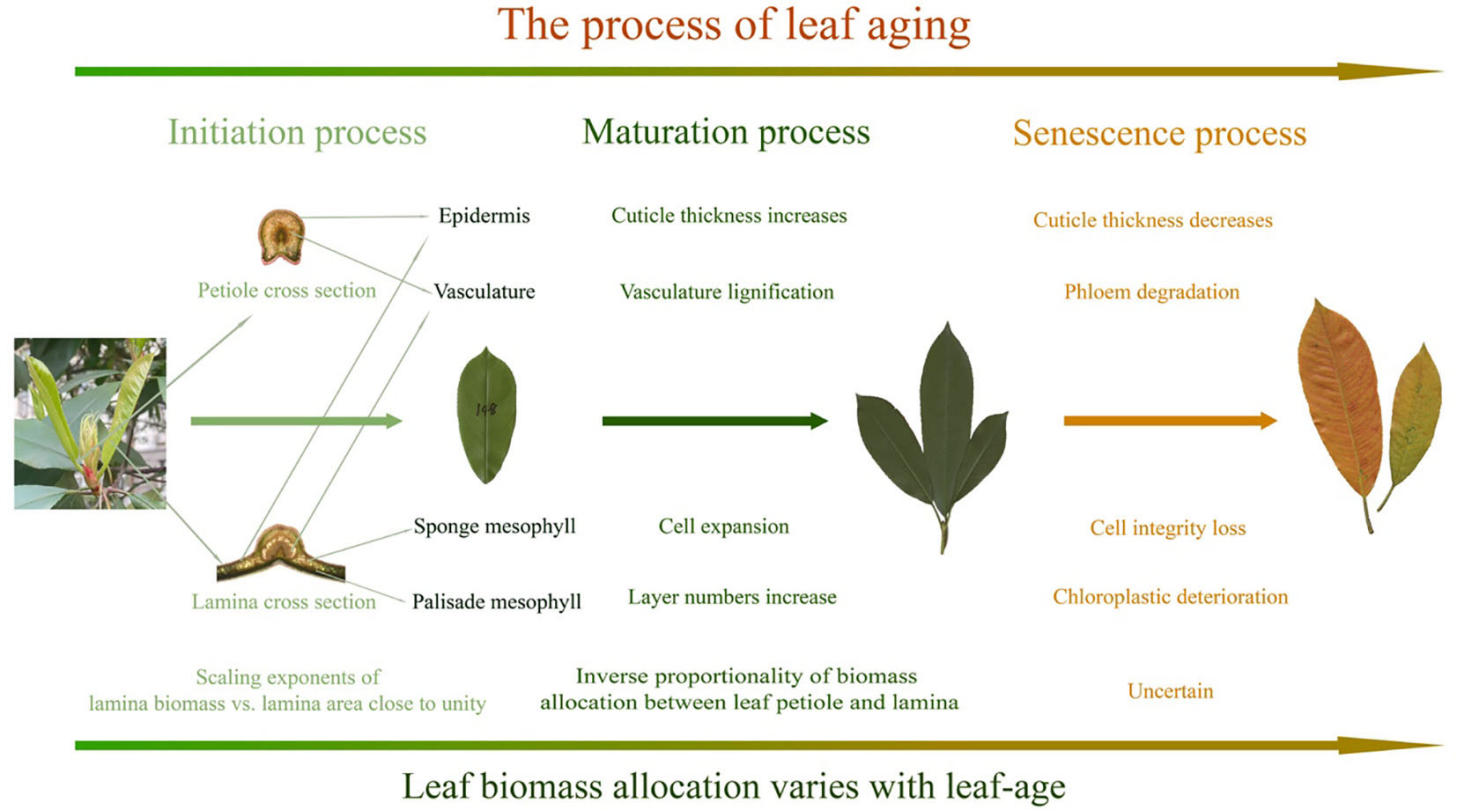
Summary of the effects of leaf-age on leaf anatomical traits and scaling exponents. The illustrated maturation processes are based on prior findings.

The foregoing caveats are important because environmental factors are known to affect leaf growth and interact with ontogeny (Hudson et al., 2011; Reich et al., 2014), such as the noticeable shift that is observed in the data collected between the second and fourth months, which might result from increasing temperature or precipitation (**Figures 2**, **3**). Environmental factors can also result in temporal changes in leaf biomass allocation patterns over the lifespans of leaves. For example, older leaves can be shaded by new cohorts of leaves, which can alter light-interception, leaf temperatures, and rates of evapotranspiration. Notably, overshadowing was not significant in our *P. serratifolia* leaf samples, as the new leaves emerged in the spring and were sampled from the upper canopy. Nevertheless, given the limited research on the effects of leaf-age on leaf biomass allocation patterns, additional studies are required across diverse species and different environmental contexts before any definitive conclusions can be drawn (**Figure 5**). In addition, the ratio and scaling approaches for describing leaf biomass allocation are based on different assumptions and theories, making the choice of whether to use fresh or dry biomass as a representation of organ biomass uncertain. To address these uncertainties, we discuss these issues separately in the following three sections.

### Ratio and scaling approaches, and the choice of fresh or dry as a representation of biomass

The difference in the results emerging from the use of the ratio and scaling approaches should be a key consideration when designing a research program (Cheng et al., 2015; Li et al., 2022). The ratio-based approach has the advantage that it reveals plant biomass allocation as a simple proportion or percentage between any two variables of interest (Wright et al., 2005; Poorter et al., 2012), whereas the scaling approach has the advantage of capturing any non-linear or linear relationship between two variables while adjusting for size or age, i.e., provides a simple way of summarizing proportional and size-dependent phenomena (Niklas, 1994; Milla and Reich, 2007; Niklas et al., 2007; Guo et al., 2022).

This study shows that the goodness of fit of the lamina fresh mass vs. lamina area scaling relationship is consistently more statistically robust than that of lamina dry mass vs. lamina area among each of the eight different leaf-age groups. The same is true for the petiole fresh mass vs. lamina area scaling relationship compared to that of petiole dry mass vs. lamina area. This phenomenon highlights the superiority of fresh mass over dry mass in describing leaf biomass allocation with the scaling approach, as previously demonstrated (see Huang et al., 2019; Liu et al., 2020; Guo et al., 2022).

Nevertheless, the foregoing results do not necessarily indicate that fresh mass should be substituted by dry mass under all circumstances. The choice should depend on the specific research question. For example, studies of nutrient cycling and ecosystem dynamics may benefit from the use of leaf dry biomass, as it reflects the long-term accumulation of plant biomass measured primarily in terms of carbon investments (Shipley et al., 2006). Dry biomass is also commonly used due to its practicality and ease of measurement, which makes it more suitable for large-scale studies and comparisons across different ecosystems (Wright et al., 2004; Poorter et al., 2012). On the other hand, studies of plant growth and development may require fresh biomass to accurately capture the plant’s physiological state (Shipley et al., 2005; Niinemets et al., 2007). Fresh biomass is also a more reasonable biomechanical trait because it reflects the loads that must be supported and because it correlates with turgor and therefore the stiffness of most primary plant tissues, particularly the hydrostatic tissues found in leaves (Niklas, 1991a; Niklas, 1991b; Niklas, 1992).

### Influence of leaf-age on leaf biomass allocation

Leaf-age significantly influences leaf morphology and chemical composition, such as leaf thickness and cellulose, as leaves grow older (Mediavilla et al., 2011), and the photosynthetic capacity of leaves is reported to decline gradually with age, presumably due to changes in leaf chemical composition, leaf nitrogen content, and CO_2_ diffusion limitation (Kitajima et al., 1997; Day et al., 2001; Han et al., 2008; Zhang et al., 2008). Consequently, the influence of leaf-age on biomass allocation patterns appears to persist throughout leaf ontogeny until maturation (**Figure 5**), as demonstrated by the observed variation in the numerical values of scaling exponents for both lamina and petiole biomass allocation with respect to lamina area (Pantin et al., 2012; Jiao et al., 2022), although the variation of leaf biomass allocation in the senescence process is uncertain (Lim et al., 2007; Koyama, 2018). Specifically, a significant opposite trend in the scaling exponents between the two allocation patterns during ontogeny is evident, which highlights the age-related effects on leaf biomass allocation.

The scaling exponent of leaf dry mass vs. lamina area tends to be close to unity in the first leaf-age group (**Table 2**), probably because juvenile leaves tend to allocate a larger proportion of their biomass to support area expansion and expedite photosynthesis-related processes, such as the development of chloroplasts and other pigments, thereby maximizing their ability to capture light and nutrients (Westoby et al., 2000). Changes in the numerical values of scaling exponents likely reflect the accumulation of secondary cell wall materials and lignification (e.g., xylem and phloem fibers) during maturation. For example, the maturation of *Laurus nobilis* leaves can be described as a “hardening process” that entails the development of the vascular tissues, and the thickening and lignification of the cell walls in the bundle sheath extensions and the epidermis (Fasseas and Akoumianaki-Ioannidou, 2010; Schuetz et al., 2014). This phenomenology, which also occurs in petioles as a consequence of the vascular tissues within them, is partly responsible for the increase in lamina dry mass per unit area (LMA) and lamina thickness with age, which is reported to correlate with increases in the concentrations of Ca and lower concentrations of N, P, K, and Mg on a dry mass basis (Mediavilla et al., 2011). Typically, a higher LMA also corresponds with thicker palisade layers, which helps to maximize overall light absorption at greater depths within the mesophyll (Coble and Cavaleri, 2017). Therefore, the scaling exponents governing the relationship between lamina mass and area numerically increase (Niinemets, 2001; Westoby et al., 2002).

Despite similar developmental patterns, the vascular development within the lamina differs from that within the petiole, especially in larger leaves with higher hydraulic resistance (Sack et al., 2004; Pantin et al., 2012). The extra leaf lamina construction demands together with biomass constraints during growth (Niinemets et al., 2004; Niinemets et al., 2006) may necessitate the prioritization biomass allocation to the petiole for increased transport capacity (Filartiga et al., 2022). Regardless of the cause, the scaling exponents of petiole biomass versus lamina biomass numerically decrease with leaf-age, indicating a shift in biomass allocation from the lamina to the petiole as leaves reach their full maturity (see **Supplementary Figure S4**). This observation is consistent with prior studies, e.g., petiole mechanical stiffness increases with leaf-age, as demonstrated in a comparison between young (May) and mature (August) leaves of *Populus tremuloides* within a single growing season (Niklas, 1991c).

### Taking leaf-age into account when assessing environmental effects

Temperature and precipitation are often reported to be the most important environmental factors affecting plant growth, thereby significantly affecting nutrient cycling, productivity, ecosystem fluxes, and other key plant and ecosystem processes (Kulmatiski and Beard, 2013; Hatfield and Prueger, 2015). Therefore, it is not surprising that leaf biomass allocation patterns to different organs or tissues are also significantly affected by temperature and other environmental conditions (Mediavilla et al., 2014; Reich et al., 2014). Two common leaf functional traits that respond to temperature are LDM and *A*, both of which are reported to increase with increasing temperature. LMA has been shown to respond plastically to abiotic variables such as light, temperature, water and nutrient availability, and atmospheric composition (Niinemets, 2001; Wright et al., 2005; Poorter et al., 2009). Thus, dynamic but predictable environmental conditions exert an influence on leaf biomass allocation patterns (Poorter et al., 2012).

However, as noted, the data reported here indicate that the “environmental effect” on the numerical value of biomass scaling exponents is statistically negligible, although the data presented here are limited both in leaf-age sample size (*n* = 8) and taxa (*n* = 1). The limited data also indicate that an “environmental effect” may be overestimated if leaf-age is not considered. At the site examined in this study, ambient temperature and precipitation gradually increase during the rainy season, especially in July and August. Likewise, the scaling exponents of lamina biomass vs. *A* numerically increased and the petiole biomass vs. *A* decreased in this period. However, after that period, the numerical values of scaling exponents are largely invariant and insensitive to changes in temperature and precipitation, even though the temperature and precipitation varied oppositely. This behavior is interpreted to indicate that scaling exponents are not responsive to changes in environmental conditions, which contradicts some previous studies (Pan et al., 2013; Thakur et al., 2019). However, additional research is clearly required to track in considerably greater detail the extent to which biomass vs. area scaling relationships respond to changes in environmental conditions, particularly during the early expansion and maturation of laminae (Westoby et al., 2022).

This caveat is particularly important because an increase in leaf biomass must be viewed as an ontogenetic process that is tied to lamina area expansion and structural maturation of both lamina and petiolar tissues. With increasing leaf-age, mechanical and hydraulic tissues are ontogenetically modified altering the biochemical and mechanical properties of cell primary and secondary walls (Wu et al., 2021). Research has also shown that the leaf circadian clock exerts control over biomass allocation patterns and that there is an asynchrony between the young leaves and older leaves (Pantin et al., 2012). Therefore, it is essential to accurately determine the age of leaves, when using either ratio or scaling methods to describe biomass allocation patterns. In passing, it is worth noting that leaf-age also affects other leaf traits. For example, maximum photosynthetic rates and nitrogen content are reported to decrease with leaf-age (Ishida et al., 1999; Han et al., 2008; Oikawa et al., 2008; Wang et al., 2015). Hence, a variety of important processes other than biomass allocation patterns are affected by leaf-age.

## Conclusions

The data emerging from this study highlight an inverse proportional allocation between leaf petiole and lamina, and the significant influences of leaf-age on the relation of both lamina and petiole mass to the lamina area during leaf maturation. The data also yield different insights when subjected to two different approaches to quantity biomass allocation patterns (i.e., the ratio approach and the scaling approach). Based on these analyses, we recommend that (i) both the ratio and scaling approaches can be applied when analysing the relevant data, and (ii) that leaf-age should be included as an important variable of interest when evaluating biomass allocation patterns. Given the significant changes in the numerical values of petiole and lamina scaling exponents as a function of leaf maturation, this work demonstrates the necessity of an ontogenetic perspective when exploring biomass allocation patterns.

## Data availability statement

The original contributions presented in the study are included in the article/**Supplementary Material**. Further inquiries can be directed to the corresponding authors.

## Author contributions

XG: Formal analysis, Writing – original draft. JS: Writing – review & editing. PS: Formal analysis, Methodology, Writing – review & editing. YJ: Formal analysis, Investigation, Writing – original draft. QM: Writing – review & editing. JX: Writing – review & editing. KJN: Formal analysis, Methodology, Writing – review & editing. 
