# Supplementary material for: Leaf-age and petiole biomass play significant roles in leaf scaling theory
Source: Front Plant Sci. 2023 Dec 21;14:1322245. doi: 10.3389/fpls.2023.1322245 (PMC10764501; doi:10.3389/fpls.2023.1322245)
Supplement: Supplementary file 1 [file DataSheet_1.docx]

Supplementary Material

**Leaf-age and petiole biomass play significant roles in leaf scaling theory**

Xuchen Guo^1^, Julian Schrader ^2^, Peijian Shi^1,^*, Yabing Jiao^1^, Qinyue Miao^1^, Jianhui Xue^1,3^, and Karl J. Niklas^4,^*

**Figure Legends**

**Figure S1**. Examples of the newly emerging leaves on trees of *P. serratifolia* that emerge only in the spring and sit at the top of the branches.

**Figure S2**. Correlation test between the estimated mean scaling exponents of LDM vs. *A* and monthly mean temperature (A); between scaling exponents of LFM vs. *A* and monthly mean temperature (B); between the scaling exponents of PDM vs. *A* and monthly mean temperature (C); and between the scaling exponents of PFM vs. *A* and monthly mean temperature (D). *r* is the correlation coefficient for the scaling exponents and leaf-ages groups, and *P* is the significance test parameter. The monthly mean temperature corresponds to those in Table 1

**Figure S3**. Correlation test between the estimated mean scaling exponents of LDM vs. *A* and monthly precipitation (A); between scaling exponents of LFM vs. *A* and monthly precipitation (B); between the scaling exponents of PDM vs. *A* and monthly precipitation (C); and between the scaling exponents of PFM vs. *A* and monthly precipitation (D). *r* is the correlation coefficient for the scaling exponents and leaf-ages groups, and *P* is the significance test parameter. The monthly precipitation corresponds to those in Table 1

**Figure S4**. Comparisons of the scaling exponents (α-values) of PDM vs. LDM (A), and of PFM vs. LFM (B). Each boxplot was obtained from 3000 bootstrap replications. The lowercase letters a–c on the top of each box denote the significance of the difference in the scaling exponents between any two leaf-ages at a 0.05 significance level. *r* is the correlation coefficient for the scaling exponents and leaf-age groups, and *P* is the significance test parameter. Leaf-age codes correspond to those in Table 1.

**Figure S5**. Non-linear regression of the scaling exponents (α-values) of LDM vs. *A* and leaf-age (A); between scaling exponents of LFM vs. *A* and leaf-age (B); between the scaling exponents of PDM vs. *A* and leaf-age(C); and between the scaling exponents of PFM vs. *A* and leaf-age (D). The lowercase letters a–c on the top of each box denote the significance of the difference in the scaling exponents between any two leaf-ages at a 0.05 significance level. *P*_1_is the significance test parameter of *x*. *P*_2_ is the significance test parameter of *x*^2^. Leaf-age codes correspond to those in Table 1.


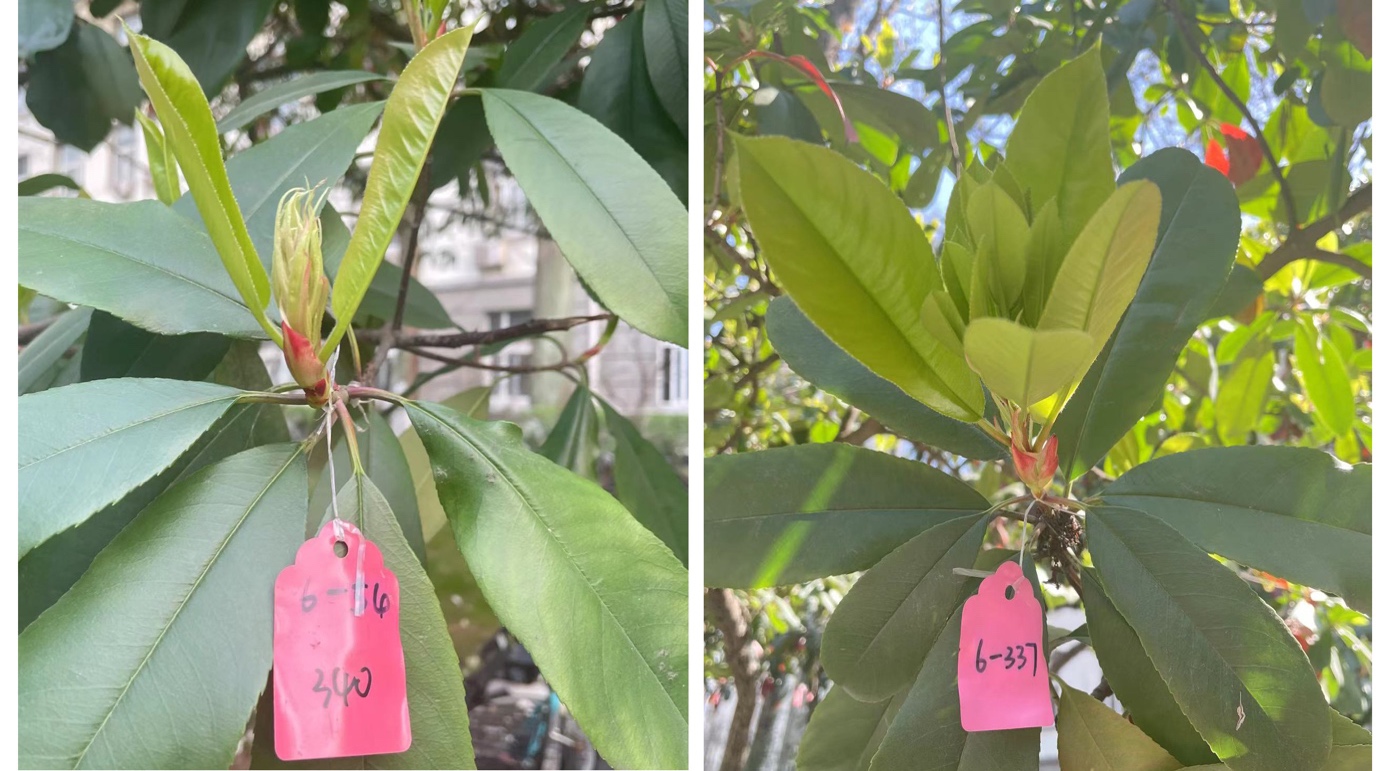


**Figure S1**

**
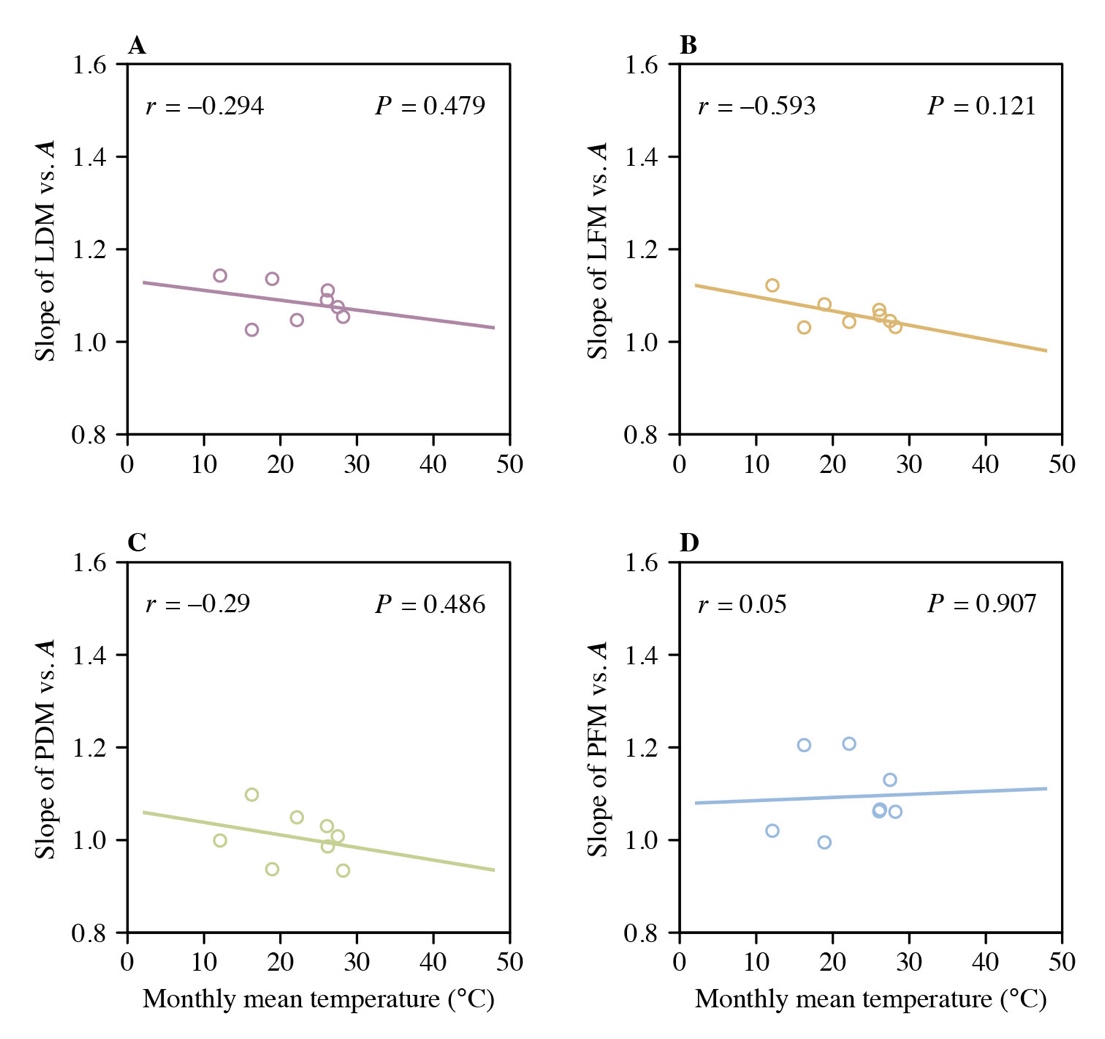
**

**Figure S2**

**
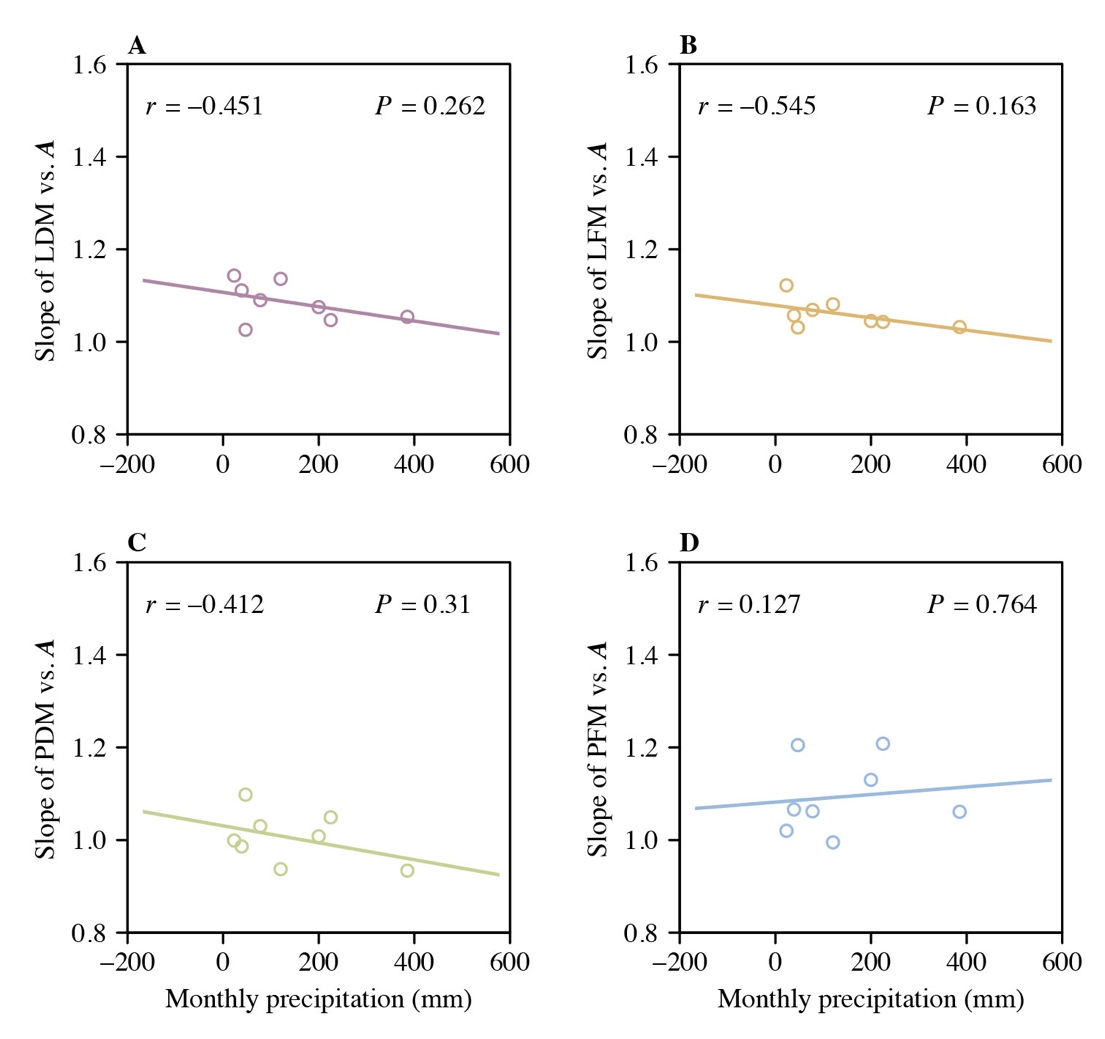
**

**Figure S3**

**
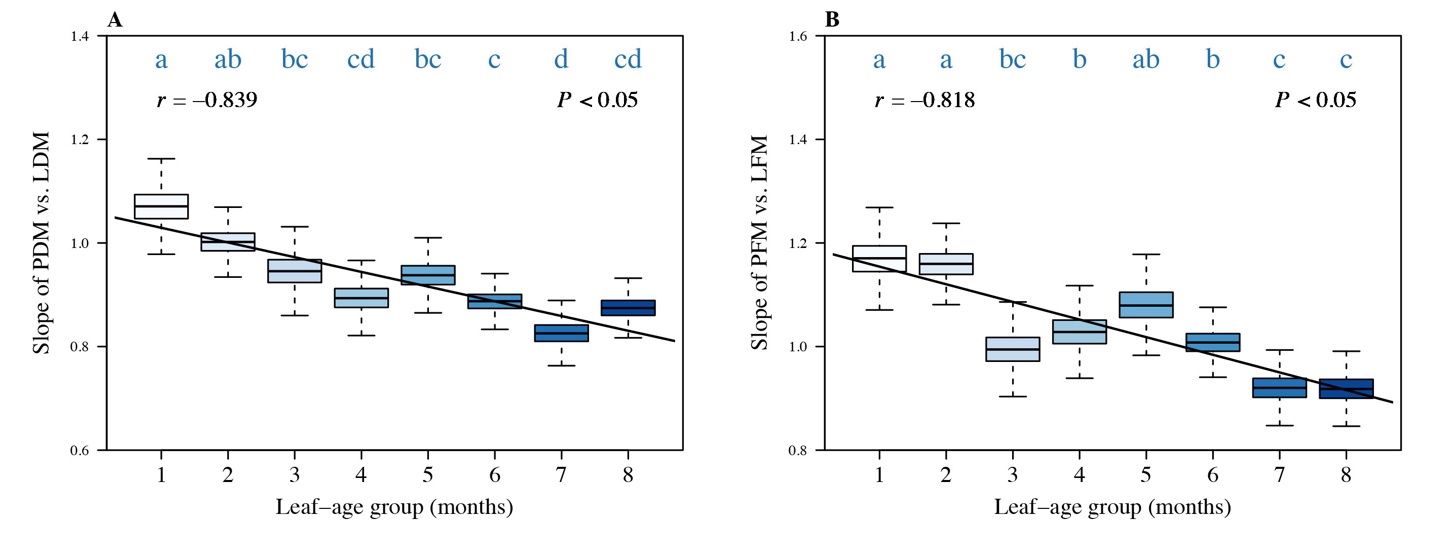
**

**Figure S4**

**
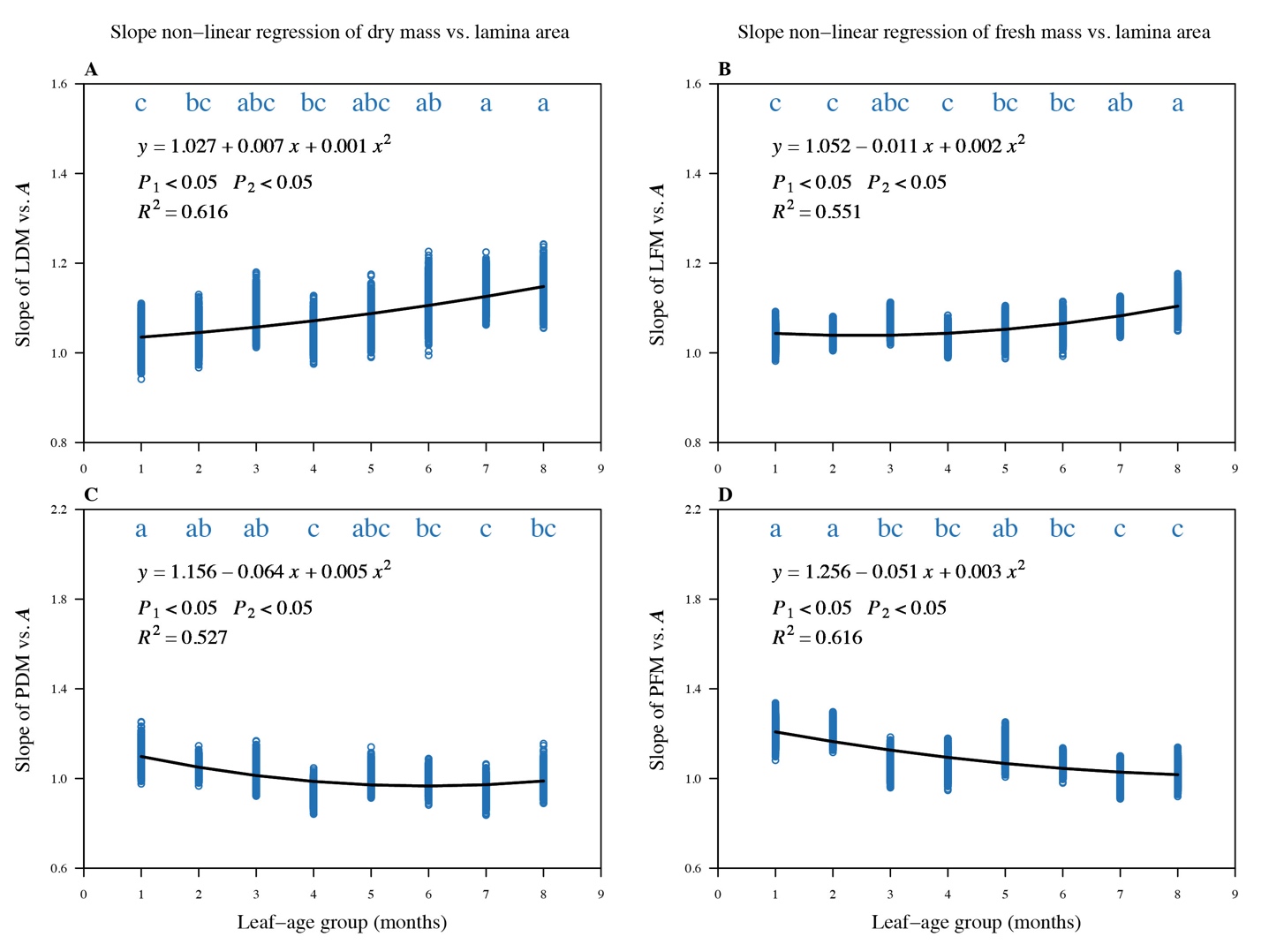
**

**Figure S5**
